# A New Method for the Detection of Neutralizing Antibodies against Mumps Virus

**DOI:** 10.1371/journal.pone.0065281

**Published:** 2013-07-05

**Authors:** Keita Matsubara, Motoko Fujino, Kaoru Takeuchi, Satoshi Iwata, Tetsuo Nakayama

**Affiliations:** 1 Department of Pediatrics, Hiroshima Prefectural Hospital, Minami-ku, Hiroshima City, Hiroshima, Japan; 2 Department of Pediatrics, Saiseikai Central Hospital, Minato-ku, Tokyo, Japan; 3 Department of Infection Biology, Division of Biomedical Science, Faculty of Medicine, University of Tsukuba, Tsukuba, Ibaraki, Japan; 4 Center for Infectious Diseases and Infection Control, Keio University, School of Medicine, Shinjuku-ku, Tokyo, Japan; 5 Laboratory of Viral Infection, Kitasato Institute for Life Sciences, Minato-ku, Tokyo, Japan; Centers for Disease Control and Prevention, United States of America

## Abstract

Neutralization test is the most reliable method of evaluating immunity against viral diseases but there is no standard procedure for mumps virus, with tests differing in the infectivity of the challenge virus, 50% plaque reduction or complete inhibition of cytopathic effects (CPE), and usage of complement. A reliable, easy, and simple neutralization test for mumps virus was developed in this study. A recombinant mumps virus expressing GFP was generated as a challenge virus. Complement was added to the neutralizing mixture at 1∶200 when stocked serum samples were used. Neutralizing antibody titers were expressed as the reciprocal of the highest dilution that did not exceed two-fold of FU values (GFP expression) of the cell control wells. A total of 1,452 serum samples were assayed by inhibition of GFP expression in comparison with those examined by conventional 100% inhibition of CPE. 1,367 (94.1%) showed similar neutralizing antibody titers when examined by both methods. The GFP expression inhibition assay, using a recombinant mumps virus expressing GFP, is a simple and time- saving method.

## Introduction

Mumps virus is a single-stranded negative sense RNA virus, belonging to the genus *Rubulavirus* of the family *Paramyxoviridae*. The mumps virus genome encodes seven major proteins in the following gene order: nucleocapsid (N), phospho (P), matrix (M), fusion (F), small hydrophobic (SH), hemagglutinin-neuraminidase (HN), and large (L) protein genes [1]. V and I proteins are also produced from the P gene. There are two envelope glycoproteins, F and HN. The HN protein is involved in the virus attachment to sialic acid receptors on the surface of host cells. This leads to a conformational change of HN which induces further conformational change of the F protein in the cascade reaction of cell fusion [1], [2]. Thus, mumps virus infection is initiated by the F and HN proteins, and neutralizing epitopes are located on these proteins [3], [4].

An acute infection of mumps virus is characterized by self-limiting demonstrable swelling of the parotid glands with tenderness and several complications have been reported following parotitis, including aseptic meningitis, deafness, orchitis, and pancreatitis [1], [5]. Mumps virus circulates throughout the world, and genotype classification of the wild type is useful for identifying the pathway of transmission [6]. Recently, circulating mumps virus strains have been divided into 12 genotypes from A to N (excluding E and M) based upon the sequence diversity of the SH gene [7], [8]. Currently circulating strains in Japan were divided into four genotypes, B, G, J, and L [9].

The isolation of mumps virus is essential for the diagnosis of patients and for monitoring the antigenicity of wild circulating strains. The efficiency of virus isolation depends mainly upon the infectious viral load in clinical samples and the sensitivity of the cells used for isolation. Vero cells have been used, but isolation is not always successful because of the low viral load, timing of sample taking, and transportation. Several serological tests have been employed for the diagnosis of mumps virus infections and, among them, the enzyme-linked immuno-assay (EIA) was used to detect IgM antibodies for diagnosis and IgG EIA to investigate immune status [10], [11]. EIA antibodies did not reflect protective immunity and a neutralization test is the most sensitive way to predict protective immunity [12], [13]. Neutralization tests take a long time to obtain results and involve several complicated procedures [14], [15]. The sensitivity of neutralization test was enhanced when complement was added [15]. Recently, the addition of complement was found to lead to deposition on the surface of viral particles bound with antibodies and destroyed the structure of mumps virus during the neutralization reaction [16]. Thus, the presence of complement seems to be essential for neutralization testing against mumps virus. In this study, a recombinant mumps virus expressing green fluorescent protein (GFP) was generated and the requirement for complement was examined using fresh and stocked sera.

## Materials and Methods

### Mumps Virus Strain

**Figure 1:**
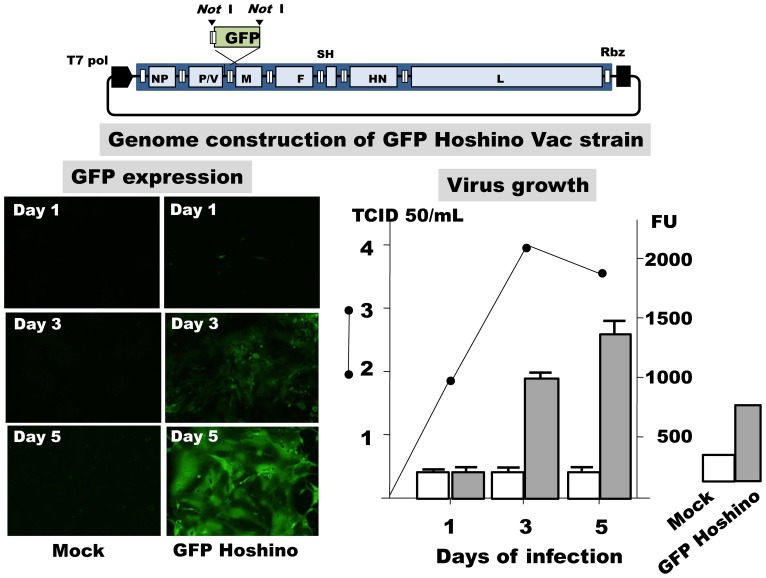
Genome construction of the recombinant mumps Hoshino strain expressing GFP and expression of GFP. Vero cells were infected with GFP Hoshino mumps strain at m.o.i. = 0.02 and subjected to experiments for GFP expression with fluoro EIA and microscopic examination on day 1, 3 and 5 of infection in comparison with mock-infection. Infectivity was assayed in culture supernatants on day 1, 3, and 5 of infection.

The Hoshino vaccine seed strain KO3 was developed by attenuation through 22 passages in chick embryonic cells from a wild-type mumps virus isolated in 1972 [17]. Full-length cDNA was constructed from KO3 Hoshino. The GFP sequence was inserted between the P/V and M genes (Fig. 1). GFP Hoshino was recovered from 293 T cells transfected with N, P, and L expression plasmids, and full-length cDNA under the control of T7 RNA polymerase [18]. Monolayer of Vero cells was infected with GFP Hoshino at m.o.i = 0.01 and culture fluid was stocked for challenge virus.

### Virus Infectivity

Vero cells were propagated in minimum essential medium (MEM) supplemented with 5% fetal bovine serum (FBS). Infectivity was determined based on the TCID_50_ in Vero cells. The virus culture fluid was serially diluted by 10-fold and a confluent monolayer of Vero cells was infected with 100 µl of each dilution in 96-well plates. The plates were incubated for 2 h at 37°C in 5% CO_2_ and MEM supplemented with 2% FBS was added. Infectivity was determined after incubation for 7 days.

### Serum Samples

Eight serum samples obtained from healthy adults aged 23 to 58 years during a routine health check were used for the experiments after obtaining verbal informed consent. The remaining portion of the sera was used for preliminary experiments or as in-house control serum. Stocked serum samples (n = 185) were obtained to assess immunity against measles, mumps, rubella, and chickenpox among new students of the nursing school of Ashikaga Red Cross Hospital, Tochigi prefecture. The serological study was approved by the ethics committee of the hospital and verbal informed consent was obtained. Fresh serum samples (n = 1,452) obtained to evaluate immunity against measles, mumps, rubella, and chickenpox among new students in primary, junior high, and high schools, were used for routine yearly immunological assessments of infection control and to advise regarding vaccination for antibody negative pupils. The serological study was approved by the Health Care Center of Keio University. The purpose of the study was explained and written informed consent was obtained from their guardians. Serum samples were anonymously transferred to our laboratory, labeled with simplified numbers.

### Virus Neutralization Test

The fresh serum samples were divided into several aliquots and stocked at −20°C. The samples were kept at 56°C for 30 min to inactivate the complement, serially diluted by 2-fold starting from 1∶4, and mixed with the same volume of GFP Hoshino containing 100 TCID_50_ of infectious virus at 37°C for 90 min for neutralization. The mixture was placed in 96-well plates in duplicate for each dilution and 25,000 Vero cells were seeded in 0.1 ml. The plates were incubated for 7 days. In order to calculate the titers automatically, the plates were processed to detect fluorescence intensity (Fluoro-Units: FU) at an emission wavelength of 528 nm and excitation wavelength of 485 nm using a fluorescence reader, FLx800 (Bio-Tek Instruments, Vermont, USA), similar to a method used to detect measles neutralizing antibodies [19]. To evaluate the requirement of complement, various concentrations of guinea pig complement (Denka Seiken, Tokyo, Japan) were added to the neutralization mixture of serially diluted serum with challenge virus. Neutralizing antibody titers were determined as the reciprocal of the highest dilutions that did not exceed two-fold of FU values (GFP expression) of the cell control wells. Conventional neutralizing antibody titers were expressed as the reciprocal of the serum dilutions that showed 100% inhibition of CPE. Infectivity titer of the challenge virus was back-titrated in each assay, showing 50–120 TCID_50_.

### Statistical Analysis

Statistical significance in the neutralizing antibody titers was examined between two groups by the Mann-Whitney test. A co-efficient was used for the analysis of correlation between the NT and EIA titers.

## Results

### GFP Expression

GFP expression and the viral growth are shown in Fig. 1. Vero cells were infected with the GFP Hoshino strain in a 24-well plate, and culture fluids were obtained 1, 3, and 5 days later. A peak infective titer of 10^4^ TCID 50/ml was obtained 3 days after infection. Mean GFP expression (FU) is shown with 1.0 standard deviation (SD) in four wells in comparison with mock-infected wells. Mock-infected wells showed approximately 300 FU during the experiment, and GFP expression in infected wells increased to peak (1,300 FU) on day 5 of infection. Together with FU in fluoro-ELISA, fluoro-microscopic findings of CPE expansion with GFP expression are also shown in Fig. 1. Few CPE were observed on day 3 of infection and extensive cell fusion was noted on day 5. The development of CPE was closely related to GFP expression.

### Neutralizing Antibody Titers

**Figure 2:**
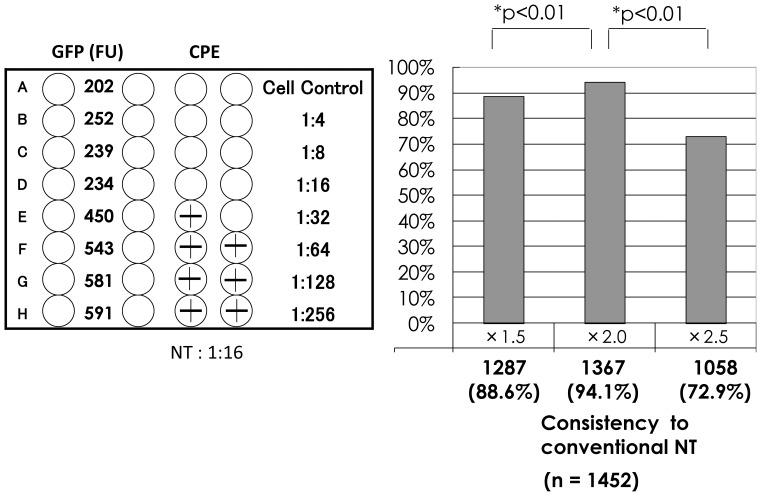
Relationship between the appearance of CPE and GFP expression. Serial two-fold dilutions from 1∶4 to 1∶256 were mixed with an equal volume of challenge virus. In the left panel, the schematic results of two neutralization methods are shown. CPE was observed in one of the two wells at 1∶32, and the conventional neutralizing antibody titer was 1∶16 by 100% inhibition of CPE. The mean FU value of the two cell control wells was 202 and that of the 1∶32 dilution was 450, showing 1∶16 of neutralizing antibody titer. Using 1,452 serum samples, the consistency of neutralizing antibody titers was compared based on different cut-off values for GFP expression: 1.5-fold, 2.0-fold, and 2.5- fold of FU values of the cell control wells.

The results of the neutralization tests are shown in Fig. 2. Serum samples were serially diluted 2-fold from 1∶4 to 1∶256, and mixed with the challenge virus. The NT assay was done in duplicate. The results for one serum sample are shown in Fig. 2. CPE were observed in one well at 1∶32 and none at 1∶16. The conventional neutralizing antibody titer was considered to be 1∶16 for 100% inhibition of CPE. The mean FU of cell control wells (mock-infected wells) was 202 FU. The mean FU of serial dilutions from 1∶4 to 1∶256 was 252 FU, 239 FU, 234 FU, 450 FU, 543 FU, 581 FU, and 591 FU, respectively. GFP expression increased to 450 FU at 1∶32 and thus the neutralizing antibody titer for the GFP expression assay was 1∶16 for inhibition of the growth of GFP Hoshino. The infective titers of the challenge virus were back-titrated, showing 50–120 TCID 50. When CPE appeared in >20% of the wells, GFP expression was >500 FU.

To evaluate the consistency of neutralizing antibody titers assayed by 100% inhibition of the appearance of CPE or GFP expression, neutralization tests for both conventional and GFP expression methods were done in 1,452 fresh serum samples. Three cut-off levels for positive GFP expression were set: 1.5-, 2.0-, and 2.5-fold increase in FU compared to cell culture controls. Among the 1,452 samples, 1,287 (88.6%) showed the similar neutralizing antibody titers when assayed by both methods using the 1.5-fold cut-off, 1,367 (94.1%) with the 2.0-fold cut-off, and 1,058 (72.9%) with the 2.5-fold cut-off. Strong similarity was noted when the cut-off was defined as a 2.0-fold of FU value in FU of the control wells.

### Effect of Heat Inactivation and Addition of Complement

**Figure 3:**
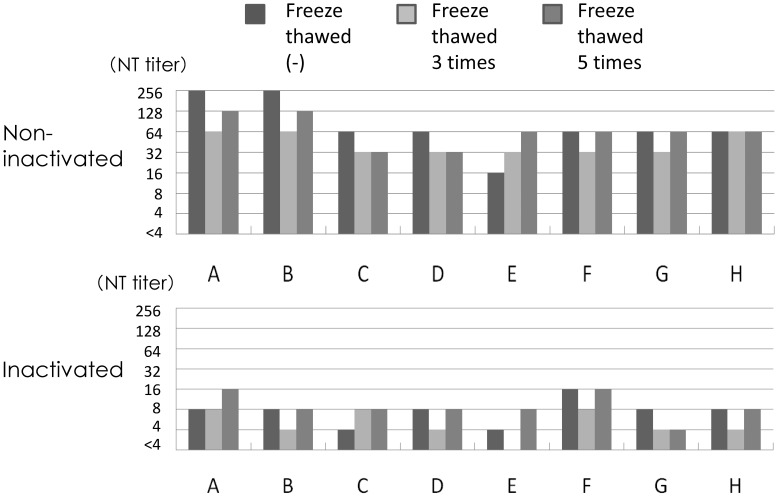
Effects of freeze-thawing and inactivation at 56°C for 30 min on neutralizing antibody titers. Upper panel shows the neutralizing antibody titers of eight fresh sera (A–H), without inactivation and after three or five rounds of freeze-thawing. Lower panel shows the results of neutralizing antibody titers after inactivation.

Eight fresh serum samples (A–H) were obtained and stocked at −80°C. Neutralizing antibody titers were examined before freeze-thawing, and after three and five rounds of freeze-thawing. The results are shown in Fig. 3. For serum A, the neutralizing antibody titer was 1∶256, 1∶64, and 1∶128, showing no significant difference within five rounds of freeze-thawing. It decreased to 1∶8 or 1∶16 after inactivation at 56°C for 30 min. The other serum showed similar results. Neutralizing antibody titers did not decrease but decreased after inactivation of the complement. Complement activity would be required for neutralization tests for mumps virus.

**Figure 4:**
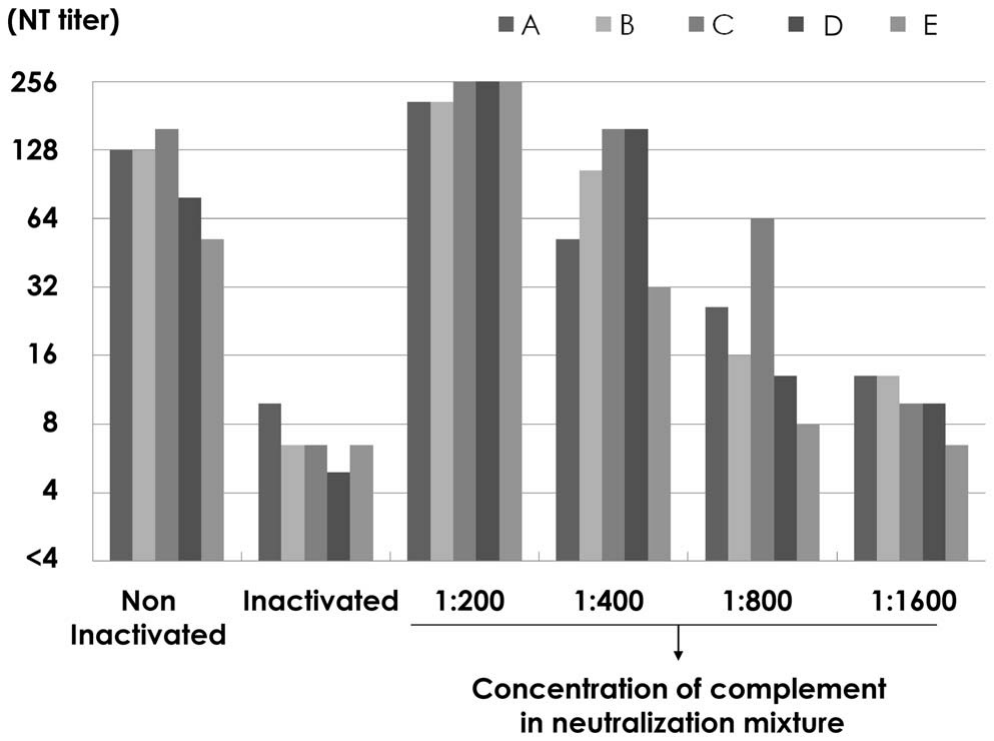
Neutralizing antibody titers of non-inactivated and inactivated sera with the addition of complement. Neutralizing antibody titers were examined in five sera (A–E) before and after inactivation. Complement was added at 1∶200, 1∶400, 1∶800, and 1∶1600 to the neutralizing mixture when inactivated sera were used. Each experiment was done in triplicate and mean titers were shown.

Five fresh serum samples (A–E) were inactivated at 56°C for 30 min. When inactivated sera were used, guinea pig complement was added to the neutralizing mixture at 1∶200, 1∶400, 1∶800, and 1∶1,600. Neutralizing antibody titers were examined and mean values for three independent assays are shown in Fig. 4. Guinea pig complement did not affect the assay system without any changes in Vero cell cultures and the addition of guinea pig complement in non-inactivated sera did not influence the neutralizing antibody titers. The titer was 1∶32–1∶128 and dropped to around 1∶8 after inactivation. The reduced neutralizing antibody titers increased to levels similar to those before inactivation when the complement was added at 1∶200 or 1∶400. Therefore, complement was added at 1∶200 to the neutralizing mixture in the subsequent experiments.

### Effect of Complement

**Figure 5:**
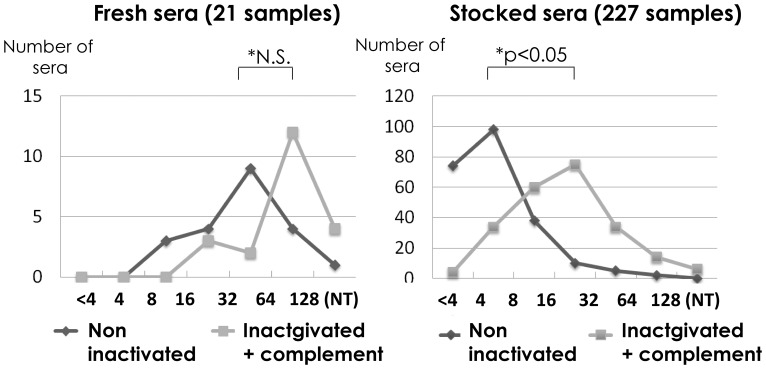
Effect of the addition of complement in 21 fresh and 227 stocked serum samples. Distribution of serum samples is shown for neutralizing antibody titers assayed without inactivation and for those assayed after inactivation with the addition of complement, using 21fresh serum samples (left panel). 227 stocked serum samples were assayed in a similar manner (right panel).

Twenty-one fresh serum samples were obtained and neutralizing antibody titers were examined for non-inactivated and inactivated sera supplemented with complement at 1∶200 in the neutralizing mixture. The results are shown in Fig. 5. The peak distribution of neutralizing antibody titers for non-inactivated samples was 1∶32 and shifted to 1∶64, showing no significant change in those with addition of complement.

As for the 227 stocked sera, neutralization tests were performed before and after inactivation with the addition of complement. 74 serum samples showed negative and 70 became positive, when assayed after inactivation with the addition of complement. The peak distribution of neutralizing antibody titers markedly shifted from 1∶4 for non-inactivated sera (98 sera) to 1∶16 after inactivation supplemented with complement (75 sera). Stocked sera were considered to lose complement activity over long periods. Therefore, the addition of complement was required when the neutralizing antibody titer was examined for the stocked sera, probably because of decreased complement activity.

## Discussion

There are several serological methods of detecting mumps antibodies. Complement fixation (CF) and hemagglutination inhibition (HI) tests are not sensitive and, in addition, HI antibodies are cross-reactive to parainfluenza virus [1], [10]. EIA has high sensitivity and specificity and is a simple procedure, but is not related to protective activity [11]. Neutralizing antibodies are associated with protective activity but the neutralizing test involves several complicated steps. Neutralization of an infectious virus and the preparation of cell cultures are bothersome and most time-consuming is the very last step to determine the appearance of CPE in 96-well plates. For micro-neutralization assays, there are two methods; 50% plaque reduction and complete inhibition of CPE. There have been several reports on neutralizing tests, concerning the evaluation of plaque reduction or inhibition of the appearance of CPE, infectivity of a challenge virus, and requirement of complement for neutralizing tests [12], [13], [14], [15].

Fujino et al. [19] reported the neutralization test for measles virus using a GFP-expressing recombinant measles virus to evaluate the neutralizing antibody titer by Fluorescent EIA reader. Here, a recombinant mumps Hoshino vaccine strain expressing GFP was developed to check the expression of GFP instead of observing the appearance of CPE or plaque counting. GFP expression was examined by a fluorescent EIA reader as fluoro-units (FU). GFP expression increased as the virus genome was transcribed after infection and was closely related to viral growth, as shown in Fig. 1. GFP expression in the cell control wells in a 96-well plate was approximately 200 FU. More than a two-fold increase in FU was considered positive for GFP expression (presence of CPE). Neutralizing antibody titers examined by GFP expression were similar to those by the conventional method for 100% inhibition of CPE (Fig. 2).

In several reports, the neutralizing step was performed without the addition of complement. Hishiyama et al. [15] reported that fresh guinea pig serum was required for neutralization tests for mumps virus. They used complement at 1∶400 dilutions in the neutralizing mixture and the addition of complement increased the neutralizing antibodies titers. Complement has several important roles in immune responses and there are three main pathways, the classical, lectin, and alternative pathways. Complement is one of the first lines of host defense and is an important part of humoral immune responses. The complement system is immediately ready to target and eliminate viral particles and to interact with specific antibodies on the surface of a virus or infected cells [20]. Complement-dependent neutralizing antibody is reported to recognize the viral glycoproteins on the virus envelope, directly related to neutralization of Vesicular stomatitis virus [21], [22], herpes simplex viruses [23], [24], and West Nile virus [25]. Cooper et al. [26] reported that the deposition of antibody and complement on the surfaces of viral particles might physically interfere with infectivity in susceptible cells due to aggregation of the viral particles. However, Friedman et al. [23] suggested that complement inhibited the infection process of HSV, indicating that it affects viral replication: virus entry, uncoating, DNA transport to the nucleus, or immediate early gene expression, not requiring particle aggregation, viral lysis, or blocking of virus attachment. Johnson et al. [16] investigated the requirement of a complement system to neutralize three closely related paramyxoviruses, Simian virus 5 (SV5), mumps virus, and human parainfluenza virus type 2 (HPIV2). HPIV2 was neutralized in a complement-independent manner but neutralization of SV5 and mumps virus proceeded through alternative pathways. However, they were neutralized by different mechanisms; C3 deposition was observed on the surface of SV5 particles, resulting in aggregates. C3 deposition was also noted on the surface of mumps virus particles but they induced virion lysis through electron microscopic findings. In this sense, the presence of complement seemed to be essential for the neutralization tests for mumps virus. When fresh sera were examined for the detection of neutralizing antibodies against mumps virus, the addition of complement did not enhance the neutralizing antibody titers and the titers were stable for 5 rounds of freeze-thawing. But the complement activity was reduced after inactivation and during long-term preservation, and the addition of complement at 1∶200 was required for neutralization tests against mumps virus.

EIA is simple and a large number of serum samples are handled without serial dilutions, which is suitable for surveillance but does not reflect protective immunity. A purified mumps virus antigen is used for the EIA antigen, and contains component proteins as well as viral particles. In our previous report, neutralizing antibodies assayed by the conventional method without complement showed a poor relationship to EIA titers. In the present study, there was again no significant relationship, with a low co-efficiency, examined by adding complement (data not shown). EIA-positive sera showed positive immune-fluorescent antibodies against the most abundant N protein [27]. Approximately 40–50% of the serum samples positive for neutralization test showed positive for immune-fluorescent antibodies against F or HN antigens, which are closely related to the infection process, attachment and cell fusion [27].

Using a recombinant mumps virus expressing GFP, the neutralization test was simplified via a reduction in GFP expression, counting automatically by fluorescent EIA reader. When stocked samples were used, complement was added at a concentration of 1∶200.
